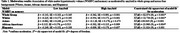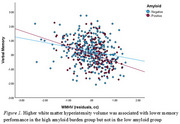# Differential associations of vascular pathology on memory performance across racial background: Moderation with amyloid positivity

**DOI:** 10.1002/alz70861_108375

**Published:** 2025-12-23

**Authors:** Shraddha Sapkota, Pauline Maillard, Audrey P. Fan, Elizabeth Rose Mayeda, Paola Gilsanz, Dan M. Mungas, Sarah Tomaszewski Farias, M. Maria Glymour, Rachel A. Whitmer, Charles DeCarli

**Affiliations:** ^1^ University of California, Davis, Davis, CA USA; ^2^ Department of Neurology, University of California, Davis, Davis, CA USA; ^3^ University of California, Los Angeles, Los Angeles, CA USA; ^4^ Kaiser Permanente Northern California Division of Research, Pleasanton, CA USA; ^5^ University of California, Davis, Sacramento, CA USA; ^6^ University of California, San Francisco, San Francisco, CA USA

## Abstract

**Background:**

Higher amyloid and vascular pathology leading to increased dementia prevalence have been reported for African Americans and Hispanics. While vascular and amyloid pathology may synergistically contribute towards cognitive decline; there is limited research on how this synergistic relationship is represented across ethnic and racial backgrounds. We examine whether vascular pathology is associated with cognitive performance across amyloid burden and whether this association differs across four groups (Whites, Asians, African Americans, and Hispanics).

**Method:**

We combined two ethnically and racially diverse longitudinal cohorts (*n*= 459; mean age= 75.22(6.21) years; 56.2% female) representing community dwelling older adults from the Kaiser Healthy Aging and Diverse Life Experiences Study and the University of California, Davis – Alzheimer’s Disease research Center. We used linear regression models to examine the association of white matter hyperintensity volume (WMHV) (representing vascular pathology) and memory. Amyloid was divided into low (<1.1SUVR, *n*=292) or high (*n*=167) amyloid groups. This analysis was repeated as stratified by Whites (*n*=180), Asians (*n*=94), African Americans (*n*=86), and Hispanics (*n*=97). Age at the time of memory assessment, sex, age difference at the time of cognitive testing and PET scan, and education were included as covariates.

**Results:**

First, we observed that both higher amyloid levels (β=‐0.237, SE=0.089, *p*=0.008) and WMHV (β=‐0.292, SE=0.071, *p*<0.001) were associated with lower memory performance. Second, higher WMHV was associated with lower memory performance in the high (β=‐0.362, SE=0.091, *p*<0.001) amyloid burden group but not in the low (β=‐0.143, SE=0.103, *p*=0.165) amyloid group (*Figure 1*, Table 1). When stratified by background, this association was only observed among Whites (β=‐0.554, SE=0.173, *p*=0.001) and African Americans (β=‐0.302, SE=0.115, *p*=0.009) but not in Asians (β=‐0.1.28, SE=0.178, *p*=0.473) and Hispanics (β=‐0.009, SE=0.170, *p*=0.657)(Table 1).

**Conclusion:**

The impact of vascular pathology on memory performance may be more prominent in those with high amyloid burden and this association may be further exacerbated in Whites and African Americans. We also observed that Asians and Hispanics were protected from this pathology suggesting the involvement of other potential genetic or lifestyle resilience factors. Older adults with high amyloid burden in specific racial and ethnic groups may benefit the most from vascular risk‐related intervention programs.